# Origins of Relative Age Effects in Youth Football—A Nationwide Analysis

**DOI:** 10.3389/fspor.2020.591072

**Published:** 2020-12-03

**Authors:** Michael Romann, Eva Rüeger, Mirjam Hintermann, Raphael Kern, Oliver Faude

**Affiliations:** ^1^Department of Elite Sport, Swiss Federal Institute of Sport Magglingen SFISM, Magglingen, Switzerland; ^2^Swiss Football Association, Muri, Switzerland; ^3^Faculty of Medicine, University of Basel, Basel, Switzerland

**Keywords:** youth sports, talent selection, talent development, waiting lists, youth football, relative age effect

## Abstract

**Introduction:** Relative age effects (RAEs) refer to the overrepresentation of players born earlier in the selection year compared to late-born players within the same age category. To date, the origins and mechanisms of RAEs are still unclear. To evaluate the development of RAEs in terms of age group and selection level, we analyzed data of all registered child and adolescent football players in Switzerland.

**Methods:** Age category, selection level, and birthdate from all licensed 101,991 Swiss child and youth football players assigned to a specific team [9,149 girls (9.0%) and 92,842 boys (91.0%); age range: 4.6–19.6 years] were analyzed. Additionally, out of 1,128 clubs, 54 clubs provided their documented waiting lists (1,224 players). Birthdate distributions were split by age category, sex, and birth quarter (Q1 = January to March, Q4 = October to December). RAEs were calculated using odds ratios (Q1 vs. Q4) with 95% confidence intervals (95% CI).

**Results:** We found small RAEs among U8 players (OR 1.44 [95% CI 1.31, 1.59]) and U10 (OR 1.24 [95% CI 1.16, 1.32]). The RAE was negligible in all other age categories, independent of gender. In children's football, 5,584 (71.3%) teams performed selections. In teams without selection, there were no obvious RAEs. However, teams with selections for the same age category showed small RAEs with an overrepresentation of Q1 athletes in the first team (OR = 1.29 [95% CI 1.24, 1.35]) and inverse RAEs with an underrepresentation of Q1 athletes in the last team (OR = 0.85 [95% CI 0.82, 0.89]). Only small RAEs were observed on the waiting lists for the U8 (OR = 1.48 [1.13, 1.95]).

**Discussion and Conclusion:** RAEs have a small, but consistent effect on participation in Swiss children's football at the grassroots level. Contrary to expectations, no inverse RAEs were found on the waiting lists. Nonetheless, first time coach selections seem to be the origin of RAEs. To protect young athletes from discrimination, RAE biases should be analyzed and eliminated at all stages of sport participation, selection, and dropout situations. Modifications to the organizational structure of sport and athlete development systems are recommended to prevent RAE-related discrimination in youth sports.

## Introduction

The practice of annual age grouping occurs throughout and across youth sport. In football (soccer), players are typically categorized into annual age groups to reduce developmental differences during childhood and adolescence. However, there is a potential gap of up to 12 months in chronological age between individuals in the same age category. Relative age effects (RAEs) are defined as the overrepresentation of chronologically older participants within one selection category (Cobley et al., 2009).

RAEs have been demonstrated in a wide variety of sports. Cobley et al. (2009), for instance, report RAEs in 14 different sports (ice hockey, volleyball, basketball, American football, Australian rules football, baseball, soccer, cricket, swimming, tennis, gymnastics, netball, Rugby Union, and golf) in male and female youth athletes from 4 years of age to adulthood. RAEs are present in all age categories and increase with age. The largest effects are observed in basketball and football. In a population-based study, RAEs in all youth athletes who were registered in the Swiss talent development program in the year 2014 were analyzed (Romann et al., 2018). A large variability in RAEs was found across different types of sports and between girls and boys. Overall, RAE was present for athletes of both sexes, with considerably larger RAE in male athletes competing at national level or in Olympic sports than male athletes competing at a lower selection level or in non-Olympic sports, respectively. Similarly, population-based analyses in France showed RAEs in basketball and football (Delorme et al., 2010, 2011). In Switzerland, population-based analyses of athletes aged 10–20 years showed RAEs in men's Swiss football and athletics (sprinting) (Romann and Fuchslocher, 2013; Romann and Cobley, 2015), as well as in women's alpine skiing, tennis, football, athletics, fencing, snowboarding, and inverse RAEs in table tennis (Romann and Fuchslocher, 2011, 2014).

RAEs can occur during the early development of youth athletes (Cobley et al., 2009; Smith et al., 2018) and may lead to a biased view of the potential of children in a particular sport. As athletes born earlier in the competition year may have more advanced physical and cognitive abilities compared to their late-born opponents, they are likely to be identified as more talented (Cobley et al., 2009; Gil et al., 2014; Wattie et al., 2015; Smith et al., 2018). Furthermore, RAEs can lead to an increased dropout from sports, with an underrepresentation of dropouts among athletes born early in the competition year and an overrepresentation among those born late in the year shown in a sample of almost 75,000 young (9–15 years) basketball players (Delorme et al., 2011). A very similar observation, i.e., an over-representation of dropouts born late in the competitive year, was reported in an analysis of more than 360,000 male French football players in the U9 to the U18 age categories (Delorme et al., 2010).

It is generally assumed that physiological growth and maturation are major underlying causes of RAEs (Cobley et al., 2009). However, there is evidence that RAEs are present before puberty and, therefore, prior to maturity-associated selection biases. Furthermore, RAEs are also found, in academia, where physical capabilities are negligible (Musch and Grondin, 2001). However, playing experience, cognitive, emotional, behavioral, motor, and social development are more likely to follow age than maturity. Therefore, such differences in development are more likely to be the underlying causes of RAEs than (differences in) maturity in athleticism (i.e., speed, power, and strength). Furthermore, several aspects, such as the popularity of a particular sport, the number of active participants, the importance of physical development and the competitive level may affect the magnitudes of RAEs (Musch and Grondin, 2001). RAEs are likely larger with high rates of participation and high selection pressure.

Additionally social agents like parents, coaches, and athletes (Cobley et al., 2009) influence RAEs. They falsely associate physical maturity with actual skill differences, and through accelerated RAEs, which have been explained with the Matthew effect, the Pygmalion effect, and the Galatea effect (Hancock et al., 2013).

However, to date, little is known about the origins of RAEs. In football, it may be speculated that RAEs increase with age and are larger in teams of higher playing levels, based on increasing selection pressure. Thus, RAEs can be expected to be smaller or even inexistent in low-level youth teams in young age groups. Accordingly, the aim of this study was to address the effect of playing levels, age groups, and selection approaches (self-selection, waiting lists, and selection by team coaches) on the occurrence of RAEs, by analyzing a population-based sample of youth football players in Switzerland.

The objectives of this study therefore were (i) to investigate RAEs in a nationwide analysis of football players (5–20 years of age), who are registered as active players assigned to an individual team in the database of the Swiss Football Association (SFA) and (ii) to evaluate the effects of different selection procedures on RAEs.

## Materials and Methods

### Participants

Following ethical approval from the Swiss Federal Institute of Sport (App. No: 082_LSP_150819), RAEs of 101,991 junior football players [9,149 girls (9.0%) and 92,842 boys (91.0%), age range: 4.6–19.6 years] participating in one of 1,128 football clubs were analyzed. Age categories ranged from under- (U) 8 to U20 for male and from U12 to U19 for female football (FF) players. Including cases where players played in two teams (different levels or different categories), we analyzed 109,469 single data points in Swiss Football Juniors' leagues during the year 2019 (Table 1).

**Table 1:** Subject characteristics and age categories in Swiss youth male and female soccer.

**Age group**	***N***	**Age**
**Mixed sex football**		
U8	3,956 (286 ♀)	6.4 ± 1.1
U10	8,316 (460 ♀)	8.1 ± 1.2
U12	23,234 (1,349 ♀)	9.7 ± 0.8
U14	23,276 (1,142 ♀)	11.5 ± 0.7
U16	17,870 (948 ♀)	13.5 ± 0.7
U18	13,250 (91 ♀)	15.5 ± 0.7
U20	9,817 (39 ♀)	17.7 ± 1.0
Subtotal U8 to U20	99,719 (4,315 ♀)	
**Female football (FF)**		
FF-12	561	10.3 ± 1.1
FF-15	2,880	12.8 ± 1.3
FF-19	1,994	15.5 ± 1.2
Subtotal FF12 to FF19	5,435	
Total ♂+♀	109,469	

*♀, female; ♂, male*.

Data were taken from the national football database with permission of SFA. In addition, all Swiss football clubs with children football's sections (U8–U12, *N* = 1,128 clubs) were contacted via email and asked whether they have (a) no waiting list; (b) anonymous waiting list, but not documented; or (c) anonymous documented waiting list. Under the term waiting list, we understand that some children sometimes cannot enter a club due to a lack of infrastructure or coaches, for example, and have to wait for a place in a football team. Out of the 1,128 clubs, 556 (49%) responded and 54 delivered their documented waiting lists, which included 1,271 players. Due to the low number of girls on waiting lists in general and of boys in the age groups older than the U12 age category, only the waiting lists of boys in the categories from U8 to U12 were analyzed (*N* = 1,224 boys).

### Procedures and Data Analysis

In Switzerland, the cutoff date for all sports is January 1st. The players were categorized into two semesters (S) and four relative age quarters (Q) according to their birth month independently of birth year (i.e., S1 = January to June; S2 = July to December and Q1 = January to March; Q2 = April to June; Q3 = July to September; and Q4 = October to December). The observed birthdate distributions were calculated for every relative age quarter. The expected birthdate distributions were obtained from the actual corresponding distributions (1999–2013) as the number of live births registered with the Swiss Federal Office of Statistics (2020). The relative age quarters of the Swiss population were as follows: Q1 = 24.5%; Q2 = 25.2%; Q3 = 26.1%; and Q4 = 24.2%. Self-selection in children's football was calculated using the distribution of the corresponding Swiss population and the distribution of registered players.

Birthdate distributions were split according to gender, age category, selection level, and birth quarter. There were up to seven selection levels possible in the same age category, from the best league to the last team of each age category within the same club. Age categories were analyzed as given by the SFA as follows: U8, U10, U12, U14, U16, U18, and U20 for the boys' categories in which girls are allowed to play, and FF-12, FF-15, and FF-19 for the only-girls' categories.

RAEs were calculated using odds ratios (Q1 vs. Q4 and S1 vs. S2) with 95% confidence intervals (95% CI). The OR for the Q1 vs. Q4 comparison was interpreted as follows: we assumed the existence of an RAE if the CI did not include 1 and interpreted an OR <1.22, 1.22 ≤ OR <1.86, 1.86 ≤ OR <3.00, and OR ≥ 3.00, as negligible, small, medium, and large, respectively (Olivier and Bell, 2013). If the OR was <1 and the CI did not include 1, this finding was interpreted as an inverse RAE. As population-based data were analyzed, inferential statistics were not applied (Gibbs et al., 2012).

## Results

The full dataset (both sexes across all age categories) shows a negligible RAE. When analyzing specific age categories, there are small RAEs in children's football (U8 and U10) and negligible RAEs in all other age categories, including FF-12 and FF-15. The FF-19 category shows an inverse RAE (Table 2).

**Table 2:** Odds ratio (OR) in each age category for distribution in birth quarters (Q1/Q4) and semesters (S1/S2), boys' and girls' categories.

**Age category**	**OR Q1/Q4**	**OR S1/S2**
U8 (*N* = 3,956)	1.44 (1.31, 1.59)	1.24 (1.15, 1.32)
U10 (*N* = 8,316)	1.24 (1.16, 1.32)	0.81 (0.78, 0.84)
U12 (*N* = 23,234)	1.13 (1.08, 1.18)	1.04 (1.00, 1.08)
U14 (*N* = 23,276)	0.98 (0.93, 1.02)	0.95 (0.91, 0.99)
U16 (*N* = 17,870)	0.99 (0.94, 1.04)	0.96 (0.92, 1.00)
U18 (*N* = 13,250)	1.00 (0.95, 1.06)	0.94 (0.90, 0.98)
U20 (*N* = 9,817)	1.06 (1.00, 1.13)	1.01 (0.96, 1.06)
Total U8 to U20 (*N* = 99,719)	1.06 (1.03, 1.10)	1.00 (0.97, 1.03)
**Female football (FF)**
FF-12 (*N* = 561)	1.10 (1.00, 1.41)	1.06 (0.94, 1.20)
FF-15 (*N* = 2,880)	0.94 (0.85, 1.05)	0.88 (0.81, 0.95)
FF-19 (*N* = 1,994)	0.84 (0.72, 0.98)	0.91 (0.81, 1.02)
Total FF-12 to FF-19 (*N* = 5,435)	0.96 (0.89, 1.04)	0.92 (0.87, 0.98)

**Figure 1:**
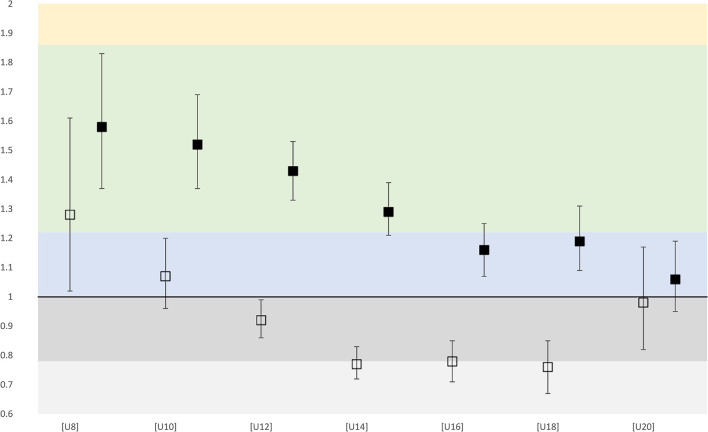
First and last level teams OR with confidence interval for each age category (male). Empty squares represent the last level teams. Full squares represent the first-level teams. Light gray color, small inverse effect; gray color, negligible inverse effect; blue color, negligible positive effect; green color, small effect; yellow color, medium effect.

Across all 1,128 clubs in childrens' football, 925 (82.0%) select multiple teams and 203 (18.0%) only select one team. Across all clubs 5,584 (71.3%) teams were built by selections and 2,250 (28.7%) were created without selection. Overall, there is a small RAE in the highest-level/first/top teams (OR = 1.29 [95% CI 1.24, 1.35]) and an inverse RAE for the lowest-level teams (OR = 0.85 [95% CI 0.82, 0.89]). Regarding specific age categories, there is a small RAE that decreases with age in the first/highest-level/top U8 to U14 teams. There is no RAE in older age categories for the first/highest-level/top teams, although OR is larger in the top/first/highest-level teams over all age categories (Figure 1). An inverse RAE with an overrepresentation of players born at the end of the selection year is found in the lowest-level teams in the U14, U16, and U18 age categories. Super, Challenge, and Promotion League Clubs show small overall RAE in their children's football teams (OR = 1.26 [95% CI 1.16, 1.36]), which decrease with age (Figure 1). None of the corresponding girl's categories show a relevant RAE.

Out of 556 children's football clubs that responded, 424 (76.3%) have no waiting list, 54 (9.7%) have a documented waiting list, 54 (9.7%) have a waiting list, but not documented, 16 (2.9%) have a periodic waiting list and 8 (1.4%) can offer an alternative program. All 54 clubs that delivered their waiting lists were included in the analysis. In total, the documented waiting lists contain *N* = 1,271 players (1,224 boys and 47 girls). The analysis of the boys' waiting lists shows a small RAE in the category U8 only (Table 3).

**Table 3:** Odds ratios on the waiting lists of categories U8 to U12 by birth quarter (Q) and semesters (S1/S2), boys' and girls' categories.

**Category**	**OR Q1/Q4**	**OR S1/S2**
U8 (*N* = 453)	1.48 (1.13, 1.95)	1.09 (0.91, 1.32)
U10 (*N* = 505)	0.84 (0.66, 1.07)	0.89 (0.75, 1.07)
U12 (*N* = 266)	0.84 (0.60, 1.20)	1.06 (0.84, 1.36)
Total (*N* = 1,224)	1.03 (0.87, 1.21)	1.00 (0.89, 1.12)

## Discussion

The main findings of this study are: (i) no relevant overall RAE, (ii) a small RAE in the youngest age group, i.e., at entry into the official football system, which decreases with age, (iii) a small effect of the current playing level of the team within the club, but not necessarily of the first adult team's playing level, and (iv) a small RAE on the waiting list of the youngest age category (U8).

### Self-Selection

The results of our study show that RAEs already exist in the youngest category for both sexes. In other words, children born at the beginning of the selection year are more likely to participate in children's football. Older children are generally biologically more mature, taller, and heavier, with more body mass for body height than younger children. This leads to advantages in athletic performance and psychological aspects, which are referred to as the maturation-selection hypothesis (Cobley et al., 2009). Therefore, it could be speculated that children born at the end of the selection year may self-select prior to playing organized football. Nationwide analyses of male French football players show similar RAEs at entry level of youth football (Delorme et al., 2010) emphasizing/reinforcing our observation. Furthermore, Smith et al. (2018), who found small RAEs (OR = 1.25 [95% CI 1.21, 1.30]) in female athletes across all sports, including football, revealed that RAE magnitude is highest in pre-adolescent (U11) and at higher competition levels. These findings indicate a self-selection effect (first hurdle) for children of both sexes prior to participating in organized sport. Furthermore, the advantage of being born at the beginning of the selection year is more pronounced the younger the children. For example, in the youngest category of children's football (U8), the age difference between two children in the selection year can be up to 12.5%, whereas this difference is reduced to about 6% in the U16 age group (Cobley et al., 2020). Fundamentally, sport participation requires opportunities to engage in sport, and the motivation to engage in such opportunities. Children born earlier in the selection year are more likely to initiate early age group participation. This may be based on a combination of early sporting experiences and encouraging interactions with others (e.g., parents and peers; Balish et al., 2014). At a young age, role models, stereotypes of the sport (women's sport vs. men's sport), popularity, positive experiences, and existing opportunities are important for sport participation, although there is a need for more in-depth examination and refinement of important correlates at various levels (intra- and interpersonal, biological, institutional, community, and policy levels; Balish et al., 2014). Therefore, peers and or parents, who are present during a child's first sports experiences, may influence the decision to join a club. Furthermore, pre-selection by parents may occur if they do not support the child's sport club participation. Therefore, a child will only join a sports club, if these two obstacles (self-selection and pre-selection by parents) are overcome.

### Coach Selection

Following club entry, further selection is conducted by coaches in over 80% of clubs, starting in the youngest age category. Coach selection seems to have a large influence on the magnitude of RAEs, with negligible RAEs due to self-selection greatly increasing as a result of first-time coach selection. This is highlighted by the fact that RAEs are higher in first teams compared to the following teams. Younger athletes may fail to meet selection requirements, as they are often biologically less developed. Therefore, their performance is likely to be poorer in age-based competition compared to athletes born earlier in the selection year. The RAEs disappear in girls in the U15 and in boys in the U14. These results differ from the results of Delorme, who found RAEs in all age categories of French basketball and football (Delorme and Raspaud, 2009). However, Delorme et al. (2010) used inferential statistics, which are not appropriate for population studies. If the data are transferred to ORs, no relevant RAEs (all ORs <1.22) were observed in U10 to adult categories for both female and male athletes. This means that RAEs at the grassroots level should be counteracted particularly in the first age categories (i.e., U8, U10).

### Underlying Mechanisms

The underlying mechanisms of RAEs in young elite athletes are well-known. In performance-oriented sport, RAEs increase with higher participation numbers, higher selection pressure, and during the growth spurt in puberty (Cobley et al., 2009). Therefore, when only assessing performance-oriented sport, peak RAEs are often associated with the ages during puberty, between the ages of 11 and 15 years. However, RAEs may be highest in children's sport and decrease consistently until the beginning of the growth spurt, which leads to a second increase in RAEs (Cobley et al., 2020). Furthermore, coaches often focus on short-term success by selecting first teams in children's football that can win matches for the club (Jimenez and Pain, 2008). However, differences in global performance, as well as success during selections of youth players mainly depend on biological and physiological variables (Coelho et al., 2010), which are inaccurate in predicting later success in adolescence and adulthood. Continued selection into better/higher teams, extra training, and elite coaching could provide first-team athletes with a performance advantage (Wattie et al., 2015). Therefore, coaches contribute significantly to the increase of RAEs in youth sports. Additionally, success in youth sport can lead to increased effort and motivation due to positive feedback from coaches, parents, and peers, and, therefore, increase performance of an athlete (Cobley and Till, 2017). Conversely, de-motivation, due to a lack of participation and selection, or the perception of a selection bias, could lead to increased levels of dropout among children and adolescents (Delorme et al., 2011; Cobley et al., 2020). Furthermore, dropouts due to demotivation are enhanced in less psychosocially supportive environments, such as larger cities (Fraser-Thomas et al., 2008). Additionally, during the talent development pathway, following previous selection, coaches (for athletes aged 10–14 years) must perform selections using a pool of players, which is already unequally distributed due to RAEs from previous selections. This process increases RAEs and dropouts even more and has been linked to the Matthew effect, the Pygmalion effect, and the Galatea effect (Hancock et al., 2013). However, there are several measures that could reduce RAEs during entry in sport, selections, and dropouts.

### Strategies to Counteract RAE Inequalities in Football

It is important to protect young athletes from age-related discrimination. To ensure this, every child should have the right to participate in sport without any age discrimination (Bergeron et al., 2015; Unicef, 2018). Therefore, RAE biases should be analyzed and eliminated at all stages of sport participation, more specifically (i) at the entry into grassroots sport participation (e.g., self-selection and club entry), (ii) in all selection procedures (e.g., waiting lists, selection into higher-performing teams), and (iii) in all dropout situations (e.g., transitions, de-selections).

Previous recommendations addressing RAE-specific inequalities are well-documented and include the creation of shorter (e.g., 6-month) and/or rotating age-group cutoff dates (Hurley et al., 2001), birthday-banding, whereby young athletes move up to their next age group on their birthday (Kelly et al., 2020), and grouping based on biological classifications (e.g., height and weight; Cumming et al., 2017). Furthermore, selection procedures at the grassroots level should be avoided. If they are indispensable, they should be improved so that no RAEs exist. However, more research and innovative approaches are needed to avoid self-selection. In regard to waiting lists, new innovative game, and training formats have been proposed that allow more players to participate (Hintermann et al., under review; Rüeger et al., 2020). During selection procedures, time points for tiers of selective representation should be delayed to post-maturation (Cobley et al., 2020). If these selections are absolutely needed, individual data can be adjusted to normal growth curves or compared with corrective adjustment procedures (CAPs) to interpret interindividual differences in performance (Votteler and Honer, 2014; Romann and Cobley, 2015; Cobley et al., 2020). Furthermore, rather than testing physical parameters, more technical and tactical competences should be tested, as they reduce RAEs and show higher prognostic validity for the transition from youth to elite adult football (Höner and Feichtinger, 2016). An improvement of the psycho-social climate that emphasizes “personal learning and development” and longer-term tracking of athletes beyond maturation have been recommended to avoid RAE-related dropouts (Cobley et al., 2014).

#### Methodological Considerations

This study has several limitations. Firstly, this study simply examines RAEs in Swiss soccer during the 2018/2019 season, which is not necessarily a reflection of the general situation over a longer period of time. Secondly, the data did not include specific match-based values (e.g., the number of matches played). Such data may provide a more complete picture of RAEs (Coelho et al., 2010). Nevertheless, this is the first study to include the complete nationwide database of both sexes across all age categories and selection levels. Therefore, this study highlights the origins of RAEs and how different selections influence the evolution of RAEs in youth soccer. Future research should address how waiting lists could be avoided and how relative age influences talent development pathways of athletes in the long run.

## Conclusion

RAEs have a small but consistent effect on participation in Swiss children's football at the grassroots level. The effect is highest at entry level and decreases from U8 to U20. Contrary to expectations, no inverse RAEs were found on the waiting lists. As U8 is the first possible club entry, RAEs could partly originate from self-selection. Nonetheless, clubs using a selection system seem to increase RAEs. The first-time coach selection at the grassroots level is the origin of RAEs and increases the magnitude of RAEs during following selection processes. To protect young athletes from discrimination, RAE biases should be analyzed and eliminated at all stages of sport participation, selection, and dropout situations. Modifications to the organizational structure of sport and athlete development systems are recommended to prevent RAE-related inequalities.

## Data Availability Statement

The raw data supporting the conclusions of this article will be made available by the authors, without undue reservation.

## Ethics Statement

The studies involving human participants were reviewed and approved by the ethics review board of the Swiss Federal Institute of Sport Magglingen. Written informed consent from the participants' legal guardian/next of kin was not required to participate in this study in accordance with the national legislation and the institutional requirements.

## Author Contributions

All authors listed have made a substantial, direct and intellectual contribution to the work, and approved it for publication.

## Conflict of Interest

The authors declare that the research was conducted in the absence of any commercial or financial relationships that could be construed as a potential conflict of interest.
